# Program of the Fourth International Dental Congress

**Published:** 1904-08-15

**Authors:** 


### PROGRAM OF THE FOURTH INTERNATIONAL DENTAL CONGRESS.

### DEPARTMENT A—SCIENCE.

SECTION I.—ANATOMY, PHYSIOLOGY, HISTOLOGY, AND MICROSCOPY. CHAIRMAN, DR. M. H. CRYER, 1420 CHESTNUT ST., PHILADELPHIA, PA.

Florestan Aguilar, Madrid, Spain: "General Anaesthesia by Somnoform." G. G. Champion, Manchester, Eng.: "Determining the Actual Path and Extent of Movement of the Mandible Condyle in the Living Subject." D. E. N. Caush, Brighton, Eng.: "Is There Uncalcified Tissue in the Enamel?" M. H. Cryer: (Subject to be announced.) W. T. Eckley: "Phylogenetic Evidence Regarding the Function of the Accessory Sinuses in Man." John E. Grevers, Amsterdam, Holland: "Anatomy of the Facial Skull—Normal and in Mouth-breathers." ————————: "Geometrical Construction of the Mandible." ————————: "Behavior of the Teeth under Polarized Light." A. Hopewell-Smith, London, Eng.: (Subject to be announced.) Eugene S. Talbot, Chicago, Ills.: (Subject to be announced.) A. H. Thompson, Topeka, Kan.: "Ethnographic Odontography; The Mound-builders and the Pre-Indian Peoples of the Mississippi Valley." J. G. Turner, London, Eng.: (Subject to be announced.) Arthur S. Underwood, London, Eng.: (Subject to be announced.) O. Walkhoff, Munich, Ger.: "Concerning the Crania of Diluvial Peoples." (Illustrated with lantern slides.)

SECTION II.—ETIOLOGY, PATHOLOGY, AND BACTERIOLOGY. CHAIRMAN, DR. R. H. HOFHEINZ, CHAMBER OF COMMERCE, ROCHESTER, N. Y. C. F. W. Boedecker, Berlin, Ger.: "Percussion in Dental Diagnosis." G. W. Cook, Chicago, Ills.: "The Effects of Chemical Agents on Bacteria with Relation to the Saliva." Samuel A. Hopkins, Boston, Mass.: "Application of the Results of Research Work to Daily Practice." W. H. G. Logan, Chicago, Ills.: "A Consideration of Some of the Etiological Factors that Produce Tissue Changes of the Alveolar Process and Overlying Soft Parts." Jos. P. Michaels, Paris, France: "Sialology; Differential Analyses; Elements of Value in Medical Diagnosis." W. D. Miller, Berlin, Ger.: "Researches Relating to Various Pathological Processes in the Teeth." Louis Ottofy, Manilla, P. I.,: "Observations on the Causes of Erosion: (*a*) Erosio Areca (*betel erosion*); (*b*) Erosio Orientalis." J. D. Patterson, Kansas City, Mo.: (Subject to be announced.) M. L. Rhein, New York, N. Y.: (Subject to be announced.) Oscar Roemer, Strasburg, Ger.: "Some Patho-histological Observations on Pyorrhea Alveolaris." D. D. Smith, Philadelphia, Pa.: "Pericemental Abscess." Eugene S. Talbot, Chicago, Ills.: "Constitutional Causes of Tooth Decay." F. Vicentini, Chieti, Italy: "Leptothrix Racemosa."

SECTION III.—CHEMISTRY AND METALLURGY. CHAIRMAN, DR. J. D. HODGEN, 1005 SUTTER ST., SAN FRANCISCO, CAL. J. P. Buckley, Chicago, Ills.: "Chemistry of Pulp-Decomposition." H. C. Carel, Minneapolis, Minn.: (Subject to be announced.) J. D. Hodgen, San Francisco, Cal.: "Chemistry and Dentistry." Hof-Zahnarzt W. Pfaff, Dresden, Ger.: "Das Aluminum und seine Anwendbarkeit in Allgemeinen." R. W. Simon, Boston, Mass.: (Subject to be announced.) Herbert L. Wheeler: "The Chemistry of Porcelain."

SECTION IV.—ORAL HYGIENE, PROPHYLAXIS, MATERIA MEDICA AND THERAPEUTICS AND ELECTRO-THERAPEUTICS. CHAIRMAN, DR. A. H. PECK, 92 STATE ST., CHICAGO, ILLS. Samuel Taylor Bassett, St. Louis, Mo.: "Application of Electro-Therapeutics to Dental Surgery." L. P. Bethel, Columbus, Ohio: "Some Results from Dental and Oral Prophylaxis." Julio Endelman, Philadelphia, Pa.: "Contribution to the Therapeutics of Post-Extraction Accidents." Richard Grady, Annapolis, Md.: "Oral Hygiene; Mastication." J. E. Hinkins, Chicago, Ills.: (Subject to be announced.) Edward Hoffmeister, Baltimore, Md.: "Materia Medica." Prof. Dr. Jessen, Strasburg, Dr. Loos,

Vienna, and Zahnarzt Georg Schlaeger: I. "Zahn-hygiene in Schule und Heer." II. Eine Wandtafel fur den Ausschauungsunterricht in der Schule in Farben, "Gesunde und Kranke Zahne." III. Eine Wandtafel, ii. Auflage auch farbig, "Die Zahne und ihre Pflage." Weston A Price, Cleveland, Ohio: (Subject to be announced.) E. Sauvez, Paris, France: "Study of the Various Means of Inducing Local Anesthesia for Extraction of the Teeth." Zahnarzt Dr. Schaeffer-Stuckert, Frankfort-on-Main, Ger.: "Paranephrin Bitsert; a New Preparation of Kidney Atrabilariun in Connection with Local Anesthetics in Dentistry." Edward Schlinkmann, Baltimore, Md.: "Electric Absorption in Therapy." C. R. Taylor, Streator, Ills.: (Subject to be announced.)

### DEPARTMENT B—APPLIED SCIENCE.

SECTION V.—ORAL SURGERY. CHAIRMAN, DR. G. V. I. BROWN, 445 MILWAUKEE ST., MILWAUKEE, WIS. Chairman's Address: G. V. I. Brown, Milwaukee, Wis.: "Oral Surgery, Its Relations to General Surgery and Dentistry." T. W. Brophy, Chicago, Ills.: "Necessity for Early Operation for Cleft Palate." T. L. Gilmer, Chicago, Ills.: "The Teaching of Oral Surgery in Our Dental Schools." H. H. Grant, Louisville, Ky.: "Solid Tumors Involving the Body or Ramus of the Inferior Maxillary Bone." J. G. Kiernan, Chicago, Ills.: "Embryogenetic, Congenital and Acquired Stomato-Neurologic Relations." A. H. Levings, Milwaukee, Wis.: "Importance and Methods of Early Diagnosis of Malignant Growths Affecting the Maxillary Bone." J. S. Marshall, San Francisco, Cal.: "Fractures of the Mandible and Their Treatment." S. E. Talbot, Chicago, Ills.: "Etiology of Cleft Palate and Hare-lip."

SECTION VI.—ORTHODONTIA. CHAIRMAN, DR. EDWARD H. ANGLE, 1023 N. GRAND AVE., ST. LOUIS, MO. Edward H. Angle, St. Louis, Mo.: "Mal-occlusion; Class II and Its Divisions." G. V. I. Brown, Milwaukee, Wis.: (Subject to be announced.) L. C. Bryan, Basle, Switzerland: "Nature as a Regulator, and Our Duty as Her Assistants." Calvin S. Case, Chicago, Ills.: "Principles and Methods of Retention in Orthodontia." M. Chiwaki, Tokio, Japan: (Subject to be announced.) Wm. Slocum Davenport, Paris, France: "Contribution to the Treatment of Short Bite and Jump Bite Cases." Robert Dunn, San Francisco, Cal.: "Mesial Position of the First Molars in Class I." John E. Grevers, Amsterdam, Holland: "Proposal for an International Nomenclature for the Various Forms of Mal-occlusion." Chas. A. Hawley, Columbus, Ohio: "Method of Determining the Normal Arch, and its Application in Orthodontia." Francisque Martin, Lyons, France: "The Correction of Deformities in Fractures of the Nose." R. Ottolengui, New York, N. Y.: "Spreading the Maxillæ *versus* Spreading the Arch." W. Booth Pearsall, Dublin, Ireland: "Irish Types of Mal-occlusion." Herbert A. Pullen, Buffalo, N. Y.: (Subject to be announced.) Jose J. Rojo, Mexico City, Mex.: "Study of the Etiology of Anomalies in Human Teeth." Dr. Schroeder, Greifswald, Ger.: "Prognathous Forms and Their Orthopedic Treatment." A. Hopewell-Smith, London, Eng.: (Subject to be announced.) J. Sim Wallace, London, Eng.: "Nasal Obstructions and Mouth-breathing, with Special Reference to Mal-occlusion of the Teeth." S. Merrill Weeks, Philadelphia, Pa.: (Subject to be announced.) Edmund Wuerpel, St. Louis: "Art." Franz Zeliska, Vienna, Austria: (Subject to be announced.)

SECTION VII.—OPERATIVE DENTISTRY. CHAIRMAN, DR. C. N. JOHNSON, MARSHALL FIELD BLDG., CHICAGO, ILLS. E. A. Bogue, New York, N. Y.: (Subject to be announced.) Jas. M. Magee, St. Johns, N. B.: "The Instrumentation and Filling of Crooked Root Canals." (Illustrated.) Sylvester Moyer, Galt, Ont.: "The Enamel and Its Consideration in Cavity Preparation." C. G. Myers, Cleveland, Ohio: (Subject to be announced.) Garrett Newkirk, Los Angeles, Cal.: "The Whole Question of Matrices and Their Uses." Frank L. Platt, San Francisco, Cal.: (Subject to be announced.) Geo. C. Poundstone, Chicago, Ills.: "The

Cement Problem in Inlay Work." M. L. Rhein, New York, N. Y.: (Subject to be announced.) Arthur Scheuer, Teplitz, Austria: "Tin Cement, Sponge Tin; Two New Filling Materials and Their Uses." E. K. Wedelstaedt, St. Paul, Minn.: "Gold and Tin." H. L. Wheeler, New York, N. Y. (Subject to be announced.)

SECTION VIII.—PROSTHESIS. CHAIRMAN, DR. C. R. TURNER, 33D AND LOCUST STS., PHILADELPHIA, PA. L. W. Baker, Boston, Mass.: (Subject to be announced.) George Brunton, Leeds, Eng. (Subject to be announced.) Reuben C. Brophy, Chicago, Ills.: "Rationale of the Use of Materials for Base-plates in the Construction of Artificial Dentures." Calvin S. Case, Chicago, Ills.: "The Mechanical Treatment of Cleft Palate." Edw. G. Christensen, Drommen, Norway: "Which is the Ideal Crown—the Banded Crown or the Crown Without Band?" B. J. Cigrand, Chicago, Ills.: "Facial Guide Lines as Taught by Artists." Bernard Frank, Amsterdam, Holland: "A New Articulator on Anatomical Principles." Hart J. Goslee, Chicago, Ills.: "Porcelain Crowns." F. H. Mamlock, Berlin, Ger. "(*a*) Ueber Porzellanstiftzahne. (*b*) Ueber Magnalium Prothesen und ihre Herstellung nach Dr. Eug. Mullerschen Gummidrucksystem." Francisque Martin, Lyons, France: "Immediate Prosthesis after the Method of Dr. Claude Martin." Joseph Nolin, Montreal, Canada: "The Decline of Estheticism in Prosthesis." B. Platschick, Paris, France: "(*a*) Influence de Fauchard sur la Prothese dentaire. (*b*) Les dents a tube en general et leur emploi special pour les pieces a gencive continue. (*c*) Contribution a letude des Couronnes." Jas. H. Prothero, Chicago, Ills.: (Subject to be announced.) Rudolph Weiser, Vienna, Austria: "Some Cases Illustrating the Possibilities of Prosthetic Dentistry." E. Lloyd Williams, London, Eng.: (Subject to be announced.) Geo. H. Wilson, Cleveland, Ohio: (Subject to be announced.)

SECTION XI.—EDUCATION. NOMENCLATURE, LITERATURE AND HISTORY. CHAIRMAN, DR. TRUMAN W. BROPHY, MARSHALL FIELD BLDG., CHICAGO, ILLS. M. Chiwaki,

Tokio, Japan: "Dentistry in Japan." Ch. Godon, Paris, France: "Educational Standards of Europe." S. H. Guilford, Philadelphia, Pa.: "Nomenclature." A. W. Harlan, New York, N. Y.: "Dental Literature." A. O. Hunt, Omha, Neb.: "The Count System of Students' Credits." Chas. McManus, Hartford, Conn.: "International Character of the Early Development of Dentistry in America." Louis Ottofy, Manila, P. I.: (Subject to be announced.) Jose J. Rojo, City of Mexico, Mex.: "Historical Annotations and Present Condition of Dental Education in the City of Mexico." B. L. Thorpe, St. Louis, Mo.: "History of American Dentistry." James Truman, Philadelphia, Pa.: "A Practical View of Education."

### CLINICS.

(Reports for the East and South have not yet been received.)

PORCELAIN: C. C. Allen, Kansas, City Mo.; W. V-B. Ames, Chicago, Ills.; E. H. Ball, Tama, Iowa.; F, E. Cheeseman, Chicago, Ills.; W. A. Coston, Ft. Scott, Kan.; W. H. Cudworth, Milwaukee, Wis.; A. W. Dana, Burlington, Iowa.; S. F. Duncan, Joliet, Ills.; Adam Flickinger, St. Louis, Mo.; V. H. Frederick, St. Louis, Mo.; H. J. Goslee, Chicago, Ills.; F. B. James, Wilton Junction, Iowa; Robt. LeCron, St. Louis, Mo.; L. A. Meyer, Oconomowoc, Wis.; J. E. Nyman, Chicago, Ills.; W. T. Reeves, Chicago, Ills.; W. H. Taggart, Chicago, Ills.; C. N. Thompson, Chicago, Ills.; J. E. Wait, Superior, Neb.: C. M. Work, Ottumwa, Iowa.

GOLD INLAYS: F. T. Breene, Iowa City, Iowa; O. H. Simpson, Dodge City, Kan.; C. N, Thompson, Chicago, Ills.; W. F. Whalen, Peoria, Ills.; C. H. Wright, Chicago, Ills.

SURGERY: T. W. Brophy, Chicago, Ills.; T. L. Gilmer, Chicago, Ills.; G. D. Moyer, Montevideo, Minn.

GOLD FILLINGS: A. G. Fee, Superior, Wis.; F. O. Hetrick, Ottawa, Can.; J. G. Pfaff, St. Louis, Mo.; C. H. Seeger, Manitowoc, Wis.; F. G. Van Stratum, Hurley, Wis.; J. W. Wick, St. Louis, Mo.

(*G. V. Black Club.*) S. Bond, Anoka, Minn.; K. E. Carlson, St. Paul, Minn.; W. R. Clack, Clear Lake, Iowa;

J. V. Conzett, Dubuque, Iowa; Wm. Finn, Cedar Rapids, Iowa; S. R. Holden, Duluth, Minn.; A. M. Lewis, Austin, Minn.; J. B. Pherrin, Central City, Iowa; G. A. Rawlings, Bismarck, N. D.; A. J. Schlueter, Aberdeen, S. D.; A. C. Searl, Owatonna, Minn.; J. F. Wallace, Canton, Mo.; E. K. Wedelstaedt, St. Paul, Minn.; R. B. Wilson, St. Paul, Minn.

GOLD CROWNS: J. G. Hollingsworth, Kansas City, Mo.; C. M. Work, Ottumwa, Iowa.

PYORRHEA ALVEOLARIS: W. H. G. Logan, Chicago, Ills.; O. H. Manhard, St. Louis, Mo.; G. R. Richter, Milwaukee, Wis.; C. R. Taylor, Streator, Ills.

EXTRACTING: J. W. Slonaker, Chicago, Ills.

MISCELLANEOUS: H. F. Cassel, St. Louis, Mo.: Striking up partial gold plate with swaged enforcement single thickness gold. W. L. Ellerbeck, Salt Lake City, Utah: Electric furnace construction. Otto J. Fruth, St. Louis, Mo.: Replantation with porcelain restoration. R. R. Johnson, Gt. Falls, Neb.: Artificial velum and obturator. Elgin MaWhinney, Chicago, Ills.: New drugs; valuable old ones; with therapeutics and indications for uses. T. W. Pritchett, Whitehall, Ills.: Bonwill method of articulating full dentures. J. B. Ridout, St. Paul, Minn.: New method of backing facings for Richmond crowns; new method of making continuous gum plate; method of putting gold corner or filling in porcelain tooth. U. A. Shannon, Lincoln, Neb.: Esthetic crowns and dummies. C. O. Simpson, St. Louis, Mo.: Demonstrating strength and durability of amalgams and cements. G. O. Sitherwood, Bloomington, Ills.: Soldering bands and fixtures; gold plating and adjusting. W. R. Smith, Pawnee City, Neb.: Prosthetic. Richard Summa, St. Louis, Mo.: Practical application of angle fracture band. S. H. Voyles, St. Louis, Mo.: Saddle bridge; pinless teeth. E. R. Warner, Denver, Col.: Masticating force of human jaws, demonstrated by appliances.

SUBJECTS NOT GIVEN: C. S. Case, Chicago, Ills.; W. D. James, Tracy, Minn.; F. E. Roach, Chicago, Ills.; Jas. Weirick, St. Paul, Minn.

